# Rapid and sensitive detection of box turtles using an electrochemical DNA biosensor based on a gold/graphene nanocomposite

**DOI:** 10.3762/bjnano.13.120

**Published:** 2022-12-06

**Authors:** Abu Hashem, M A Motalib Hossain, Ab Rahman Marlinda, Mohammad Al Mamun, Khanom Simarani, Mohd Rafie Johan

**Affiliations:** 1 Nanotechnology and Catalysis Research Centre, Institute for Advanced Studies, University of Malaya, 50603, Kuala Lumpur, Malaysia; 2 Microbial Biotechnology Division, National Institute of Biotechnology, Ganakbari, Ashulia, Savar, Dhaka-1349, Bangladesh; 3 Department of Chemistry, Jagannath University, Dhaka-1100, Bangladesh; 4 Department of Microbiology, Institute of Biological Sciences, Faculty of Sciences, University of Malaya, 50603, Kuala Lumpur, Malaysia

**Keywords:** box turtle, DNA detection, electrochemical DNA biosensor, nanocomposite, screen-printed carbon electrode

## Abstract

The Southeast Asian box turtle, *Cuora amboinensis*, is an ecologically important endangered species which needs an onsite monitoring device to protect it from extinction. An electrochemical DNA biosensor was developed to detect the *C. amboinensis* mitochondrial cytochrome b gene based on an in silico designed probe using bioinformatics tools, and it was also validated in wet-lab experiments. As a detection platform, a screen-printed carbon electrode (SPCE) enhanced with a nanocomposite containing gold nanoparticles and graphene was used. The morphology of the nanoparticles was analysed by field-emission scanning electron microscopy and structural characteristics were analysed by using energy-dispersive X-ray, UV–vis, and Fourier-transform infrared spectroscopy. The electrochemical characteristics of the modified electrodes were studied by cyclic voltammetry, differential pulse voltammetry (DPV), and electrochemical impedance spectroscopy. The thiol-modified synthetic DNA probe was immobilised on modified SPCEs to facilitate hybridisation with the reverse complementary DNA. The turtle DNA was distinguished based on hybridisation-induced electrochemical change in the presence of methylene blue compared to their mismatches, noncomplementary, and nontarget species DNA measured by DPV. The developed biosensor exhibited a selective response towards reverse complementary DNAs and was able to discriminate turtles from other species. The modified electrode displayed good linearity for reverse complementary DNAs in the range of 1 × 10^−11^–5 × 10^−6^ M with a limit of detection of 0.85 × 10^−12^ M. This indicates that the proposed biosensor has the potential to be applied for the detection of real turtle species.

## Introduction

The Southeast Asian box turtle (BT), *Cuora amboinensis*, is an endangered and protected turtle species. Due to its high value as an exotic food item and in traditional medicine, it ends up being a profitable item in the illicit wildlife trade [1]. Turtles contain active therapeutic properties and stimulating components [2–3]. The animals are captured for local use, sold as pets, utilised as foodstuffs, and in traditional Chinese remedies [4]. In addition, tortoise shells are used to make gels, soups, pills, and capsules [5].

However, these animals are hosts of several microbes and sources of heavy metals/toxins [6–7], and are prohibited from consumption by Muslims [5]. Therefore, turtle materials in food chains and medicines pose both health and social risks. Aside from that, a rising demand encourages the illicit trafficking of BT [8]. Illegal turtle meat and species exports have been detected in many countries [8–9].

Turtle species are particularly endangered: 2.7% of the species are already extinct and a total of 66.7% are threatened [10]. India, Malaysia, Myanmar, Thailand, Indonesia, Vietnam, Laos, the Philippines, Singapore, Cambodia, and Bangladesh are all homes to the BT [8]. The species was placed in the vulnerable category in Appendix II of the Convention on International Trade in Endangered Species of Wild Fauna and Flora (CITIES) and in the International Union for Conservation of Nature (IUCN) to combat illicit trade [11].

In order to avoid or decrease unlawful trafficking and adulteration of foods and medicines, there is an obvious need for a trustworthy authentication method to identify turtle species [9]. Hence, it is crucial to develop an accurate and sensitive analytical technique for the authentication of species [12]. However, the complex nature of food, various types of adulterants, and technological advancements in meat processing have made quality check extremely difficult and often pose a challenge in the authentication of animal origins [13].

Numerous analytical approaches to identify animal species in food items have been developed over time [14–15]. However, molecular biology techniques such as protein- [16] and DNA-based [17] methods are commonly used.

The structure of proteins in processed meat products is altered, reducing the accuracy of species identification in processed meals [18–19]. DNA-based technologies are thought to be more stable compared to protein-based methods [20]. A variety of DNA-based PCR techniques [21–24] is widely used for species identification. Although such techniques are selective, sensitive, and repetitive, they are time-consuming, laborious, have complex laboratory protocols [12,16], are cost-intensive [16], and cannot be used onsite. In addition, hazardous chemicals are frequently employed to PCR-based assays [25]. There is also a concern about PCR product carryover contamination due to the 10^8^-fold amplification of the target [26]. Finally, shorter DNA targets would be thermodynamically more stable than longer ones [27] and choosing a shorter target is favourable since it can withstand decomposition states [28].

To ameliorate these limitations and in order to rapidly verify the products, low-cost, highly sensitive, miniaturised, easily and onsite-applicable [29], health- and environmentally friendly, contamination-free, and shorter DNA-target-based devices are demanded for species screening. That is why the emphasis has been attributed to developing a DNA-based electrochemical biosensor comprising all the qualities mentioned above [29–30]. There are few reports available concerning the applications of impedance DNA hybridisation biosensors for the detection of a number of analytes [31–34].

Nanomaterials may significantly enhance biosensor performance, stability, repeatability, and sensitivity [35–39]. Among various nanomaterials, graphene (Gr) [40] and gold nanoparticles (AuNPs) [32] based nanocomposites are well established [41] due to their excellent performance. In particular, self-decorated AuNPs in the honeycomb-structured graphene lattice could facilitate the accommodation of a greater number of recognition probes. In addition, Gr is a nanomaterial with a large surface area, high electrical conductivity and electron transfer rate, and it can immobilise diverse molecules, which is ideal for biosensor design [37–38,42].

On the other hand, AuNPs offer outstanding characteristics such as biocompatibility, conductivity, catalytic efficiency, density, and surface-to-volume ratio [37–38,43–46]. Biomolecules such as DNA may readily modify AuNPs by adding thiol and amine groups via Au–S or Au–N links without losing their activity [38,47]. In electrocatalytic applications, the combination of carbon-based materials with metal nanoparticles has been shown to have synergistic benefits [36,48]. As a result, there are good reasons to believe that combining Gr and AuNPs in biosensing will result in a synergistic impact on electro-oxidation [30,37]. Considering these, we hypothesised that a combination of Gr and AuNPs would increase the detection sensitivity of a nanosensor.

Mitochondrial DNA (mtDNA) has numerous advantages over nuclear genomes, including increased quantity and copy number in each cell, which may be used to differentiate intra- and interspecies [49–50].

In this study, we identified and selected a unique DNA sequence in BT by comparing mitochondrial cytochrome b (cytb) gene sequences from a group of 30 species using bioinformatics tools to design a unique DNA probe. The screen-printed carbon electrode (SPCE) was modified with a nanocomposite containing Gr and AuNPs to improve detection ability and to anchor the capture probe with AuNPs. Finally, a biosensor was developed, integrating that probe sequence into a nanocomposite-modified SPCE to detect *C. amboinensis*. In addition, the developed in silico probe-based electrochemical nanobiosensor was validated in a wet-lab experiment. It was then tested to see if it could differentiate BT DNA from DNAs of other animals by using differential pulse voltammetry (DPV) using methylene blue (MB) as a redox species.

## Results and Discussion

### Design of a unique probe for BT

The identification and differentiation of different species is difficult because related species share many phenotypic and biochemical characteristics controlled by common genes. Therefore, their authentic identification remains a problem [51]. The cytb gene is highly conserved among different species and shows diversity among various unrelated populations of the same species. The use of the mitochondrial cytb gene has been shown to be a reliable and sensitive method for species identification [49]. In most vertebrates, the cytb gene is approx. 1,140 base pairs in length and demonstrates sufficient interspecies variation [50]. This is why cytb was chosen for the development of a BT-specific probe.

**Figure 1:**
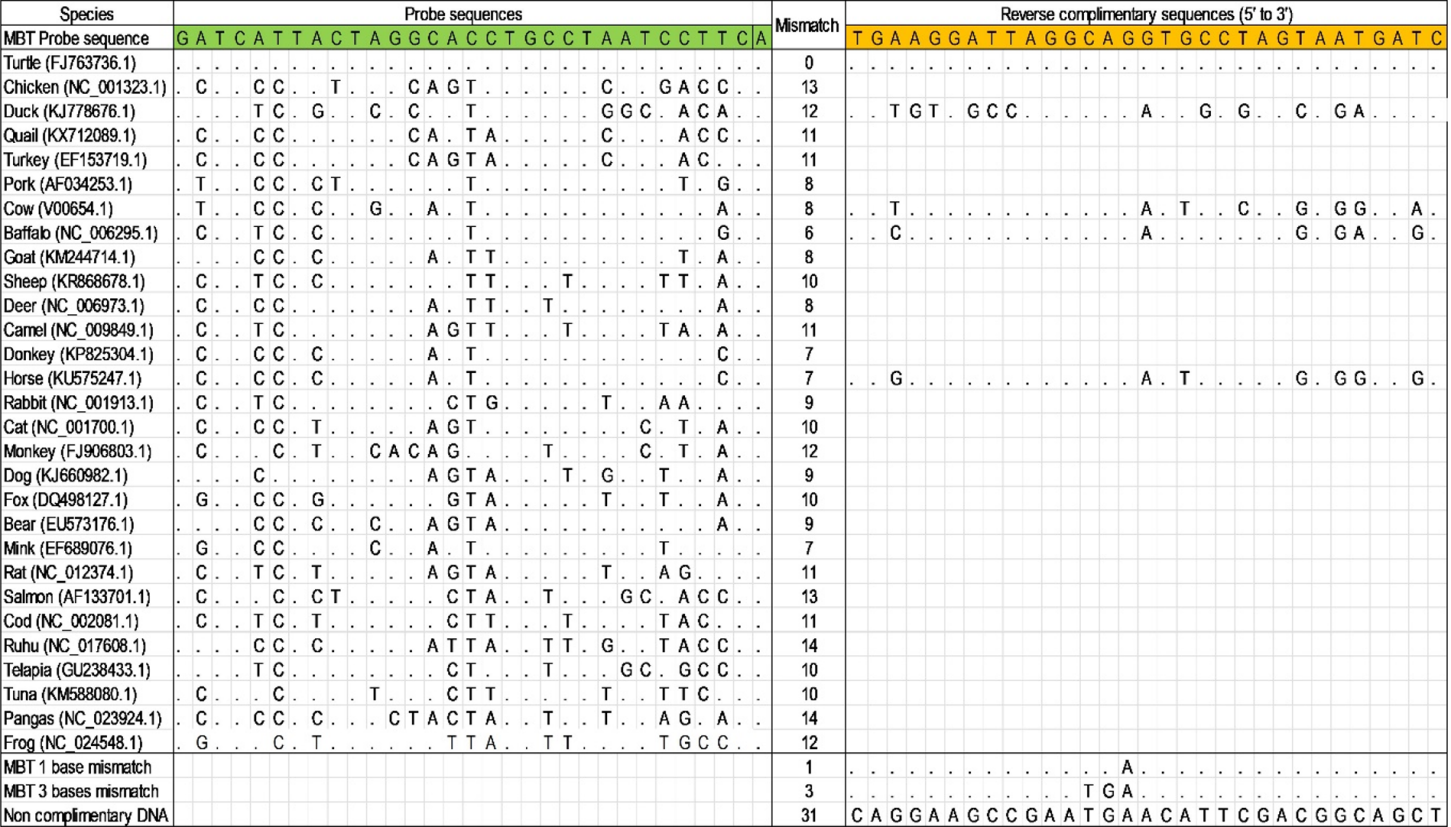
The *Cuora amboinensis* specific probe (31 bases) and corresponding aligned sequences. A, T, G, C (A, adenine; T, thymine; G, guanine; C, cytosine) stand for the corresponding nucleotides. The dot (.) indicates a match with BT, and the corresponding nucleotide alphabet indicates a mismatch with BT.

A BT-specific DNA sequence was chosen as the probe from aligned sequences with the highest mismatch with 29 nontarget species (Figure 1). From the aligned sequences, a 31 base length sequence was chosen as the probe. The selected probe sequence was matched 100% with the BT DNA and 6–14 bases (19–45%) were mismatched with nontarget species (Figure 1). The reverse complementary sequence of the probe was designed and synthesised to have 100% complementarity with the probe. Besides this, reverse complementary sequences of some common meat species, such as cow, buffalo, horse, and duck were also synthesised to compare hybridisation efficacy, and the results show discrimination of BT from other animals. The sequences of the targeted regions of all 30 species, their accession numbers, selected probe sequence, mismatch sequences, and reverse complementary sequences are shown in Figure 1.

### Characterisation of the modified screen-printed carbon electrode

#### Surface morphology and structural characterisation

**Figure 2:**
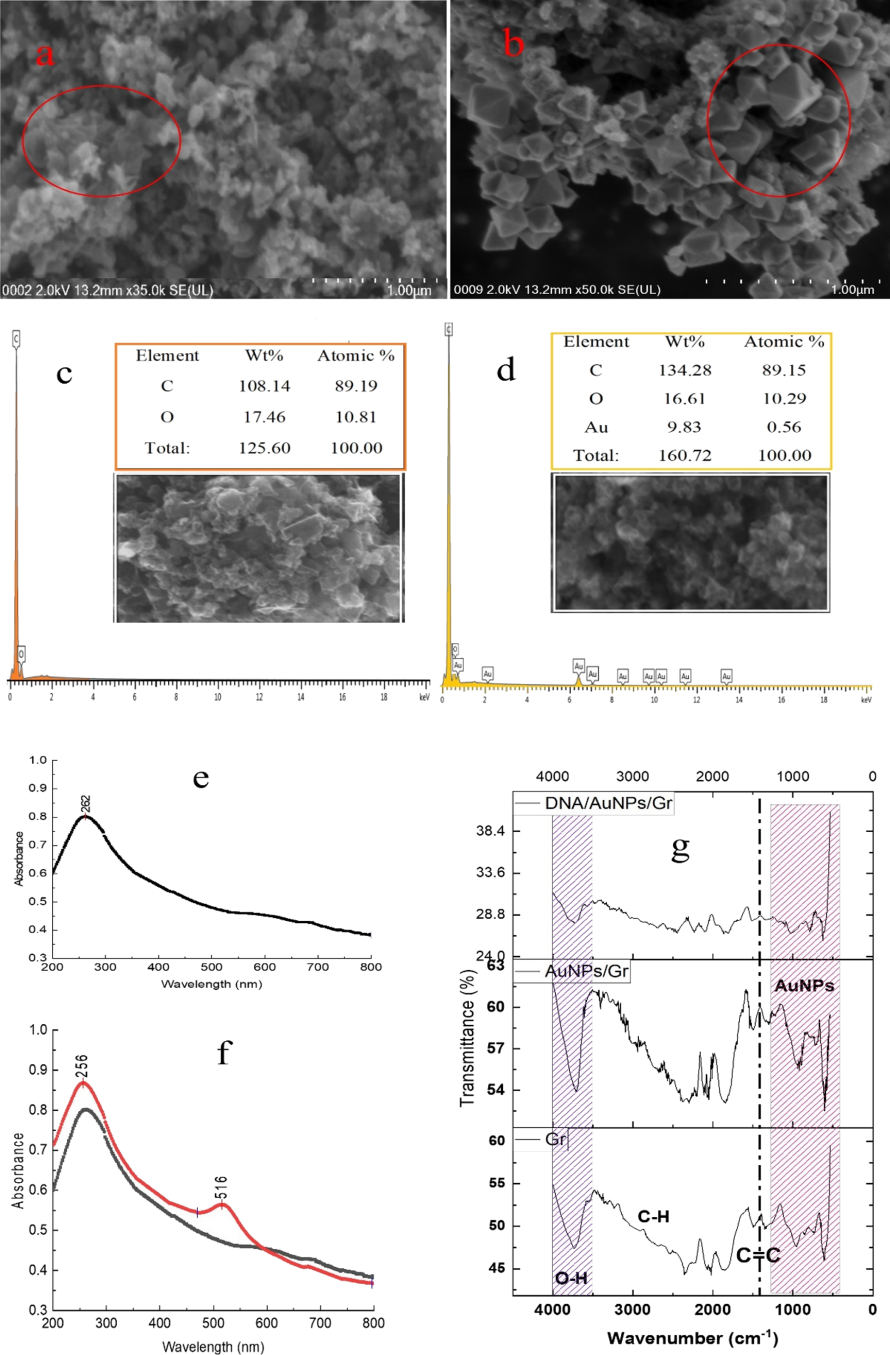
Morphological and structural characterisation of different nanoparticles and their composites. FESEM images of (a) Gr and (b) AuNPs/Gr; EDX of images of (c) Gr and (d) AuNPs/Gr; UV–vis spectra of (e) Gr and (f) AuNPs/Gr; and FTIR spectra of (g) Gr, AuNPs/Gr, and DNA/AuNPs/Gr. AuNPs were taken (33.33%, 1:3) in the AuNPs/Gr nanocomposite.

Field-emission scanning electron microscopy (FESEM) images of nanoparticles and their composites are depicted in Figure 2a–b, where the wrinkled regions represent specific patterns of different materials. In Figure 2a, the wrinkled area represents folded Gr sheets [52]. In Figure 2b, octahedron-like gold particles [53] are clearly visible in the corresponding FESEM image. The particle size of AuNPs seems to be larger than expected, which may be due to self-aggregation during the formation of the nanocomposite with graphene. The spectrum of Gr in Figure 2c obtained by energy dispersive X-ray spectroscopy (EDX) clearly demonstrates the presence of a high amount of carbon with a minimum amount of oxygen, indicating the purity of Gr. Figure 2d clearly shows the presence of carbon, oxygen, and AuNPs in the composite. The weight and atomic contribution of each of these elements in the nanocomposite were given with their corresponding EDX spectrum. The use of AuNPs–Gr as a DNA immobilisation matrix enhances the active surface area of the modified SPCE, allowing for a stronger DNA hybridisation detection signal.

UV–vis spectra were used to determine the presence of Gr and AuNPs/Gr in the composite. From the image (Figure 2e), Gr has a peak at 262 nm, which has been shifted to 256 nm (Figure 2e) in the composite, possibly due to interactions between AuNPs and Gr. Additionally, the distinct peak at 516 nm in the composite (Figure 2f) indicated the presence of AuNPs. Fourier-transform infrared spectroscopy (FTIR) was used to investigate the vibrational spectrum of the functional groups present in the nanoparticles and their composites. The FTIR spectra of Gr, AuNPs, and DNA/AuNPs/Gr were fused in Figure 2g, demonstrating the successful alteration of the spectra in several positions. A significant deviation was observed at a peak near 2357 cm^−1^. The sharpness of the Gr peaks was increased after adding AuNPs, which was reversed when DNA was added to the composite. Besides these, other peaks were also modified after the immobilisation of DNA. Supporting Information File 1, Table S1 illustrates the major peaks and their shifting after each modification of the nanocomposites.

#### Modified effective surface area of screen-printed carbon electrodes

**Figure 3:**
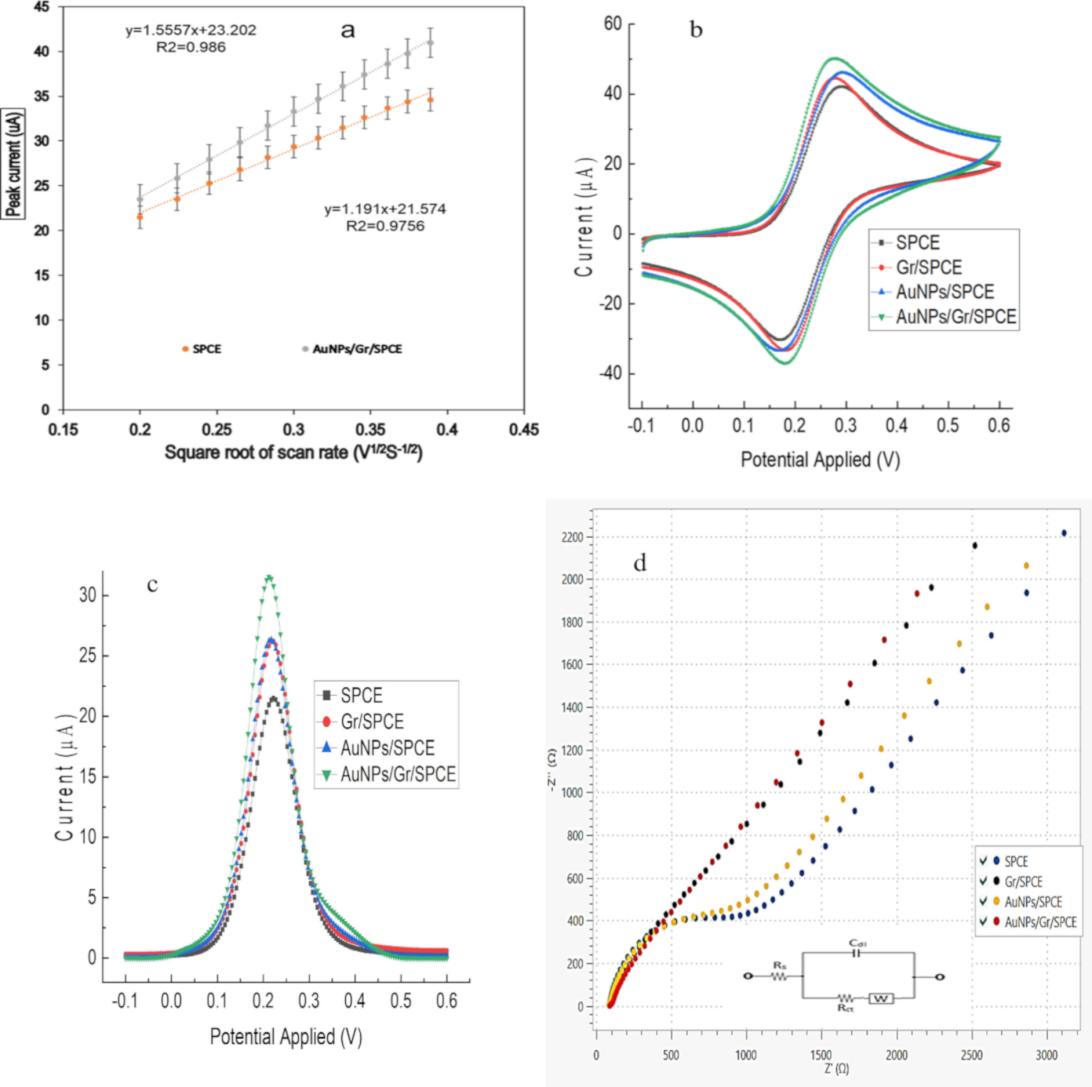
Electrochemical characterisation of various modified electrodes. (a) The relationship between the peak current and square root of the scan rate within the linear range from 40 to 150 mV for SPCE and composite- (Gr/AuNPs) modified SPCE for determination and comparison of the effective surface area. (b) CV for SPCE and modified SPCE within the potential window of −0.1 to 0.6 V at a scan rate of 100 mV/s vs Ag/AgCl. (c) DPV for SPCE and modified SPCE at a potential range from −0.1 to 0.6 V, a step potential of 5 mV, and modulation amplitude of 25 mV. (d) EIS for SPCE and modified SPCE was carried out at a frequency range from 0.10 to 100 KHz and an amplitude of 10 mV_RMS_. All experiments were carried out in a 2.0 mM K_4_[Fe(CN)_6_] solution in 0.2 M KCl.

Cyclic voltammetry (CV) was employed to measure SPCE and modified SPCE at different scan rates ranging from 40–150 mV/S with 2.0 K_4_[Fe(CN)_6_] in 0.2 M KCl. The initial geometrical surface area of SPCE was 0.11 cm^2^, which was increased to 0.204 cm^2^ after modification with Gr and AuNPs. The results indicate that the modification of SPCE with the Gr/AuNPs nanocomposite has significantly increased the effective surface area. This facilitated more nucleotide probe attachments and increased signal intensity of the biosensor. The peak current of AuNPs/Gr/SPCE linearly rose with the square root of the scan rate (v^1/2^) in the range of 0.2–0.389 V^1/2^ S^−1/2^, as shown in Figure 3a. This shows that the diffusion process regulates the electrochemical reaction of ferrocyanide. Given that the diffusion coefficient and electrolyte concentration were constant throughout all tests, the effective surface area (A) of the electrode had the greatest influence on the peak current (Ip).

#### Electrochemical characterisation of screen-printed carbon electrodes

Figure 3b shows the CV behaviour of SPCE and different modified SPCEs with 2 mM of a [Fe(CN)_6_]^3−/4−^ aqueous solution in 0.2 M KCl at a scan rate of 100 mV/S. Compared with bare SPCE, the peak currents of Gr/SPCE and AuNPs/SPCE considerably increased, indicating that Gr and AuNPs had very good conductivity and catalytic functions. The peak currents on AuNPs/Gr/SPCE were higher and more reversible when the Gr/AuNPs nanocomposite was deposited on the surface of SPCE, suggesting that the Gr/AuNPs nanocomposite was an excellent electric conductor which accelerated the electron transfer rate of [Fe(CN)_6_]^4−/3−^.

The DPV of SPCE modified with the AuNPs/Gr nanocomposite exhibited the highest peak current in 2.0 mM K_4_[Fe(CN)_6_] compared to that of the bare surface of SPCE (Figure 3c). The DPV peak current values for AuNPs/Gr, AuNPs, and Gr electrode surfaces were 31.55, 26.36, and 26.21 mA, respectively. This finding proves that the AuNPs/Gr nanocomposite is suitable for electrochemical analysis and enhances the electrocatalytic activity by facilitating electron transfer in the redox process [54].

Bare SPCE and modified SPCE surfaces were examined using electrochemical impedance spectroscopy (EIS) measurements. In 2.0 mM [Fe(CN)_6_]^3−/4−^ containing 0.2 M KCl as the supporting electrolyte, the Nyquist plots of the SPCE, Gr/SPCE, AuNPs/SPCE, and AuNPs/Gr/SPCE are shown in Figure 3d. The inset is the equivalent circuit indicating the charge transfer resistance (*R*_ct_), double layer capacitance (*C*_dl_), Warburg impedance (*W*), and the solution resistance (*R*_s_) [55]. As the Warburg impedance (which corresponds to the diffusion of the redox species) is limited at lower frequencies, the full semicircle disappeared which indicates the dominating diffusion-controlled charge-transfer process through the electrode–solution interface. Regarding the qualitative aspect, it was found that the nanocomposite-modified electrode shows a diffusion-limited charge transfer process. The bare SPCE comparatively showed lower charge transfer resistance at higher frequencies than that of individual nanomaterial-modified SPCEs, representing a limitation of electron transfer through the electrode–solution interface [56] for different electrode surfaces. The EIS plots for the modified SPCEs surfaces also reveal that the electrode–solution interfacial charge transfer process is not a purely kinetic or diffusion-controlled process. The solution resistance remains constant for almost all the electrode surfaces since they are measured in the same electrolytic medium.

After modification with individual Gr and AuNPs, the change in the Nyquist plot is relatively small, whereas it shows a significant sensitivity when modified with the AuNPs/Gr nanocomposite, which is also corroborated by CV and DPV results. These findings imply that electrode modification with AuNPs/Gr enhances electrocatalytic activity. Many researchers have reported a similar shape of the Nyquist plot when different electrodes are modified [57–59].

### Optimisation of experimental parameters

The hybridisation efficiency has a significant impact on the selectivity and sensitivity of biosensors. In order to enhance the hybridisation efficiency, hybridisation conditions of the DNA probe with the reverse complementary target were optimised to obtain an optimum DPV response.

**Figure 4:**
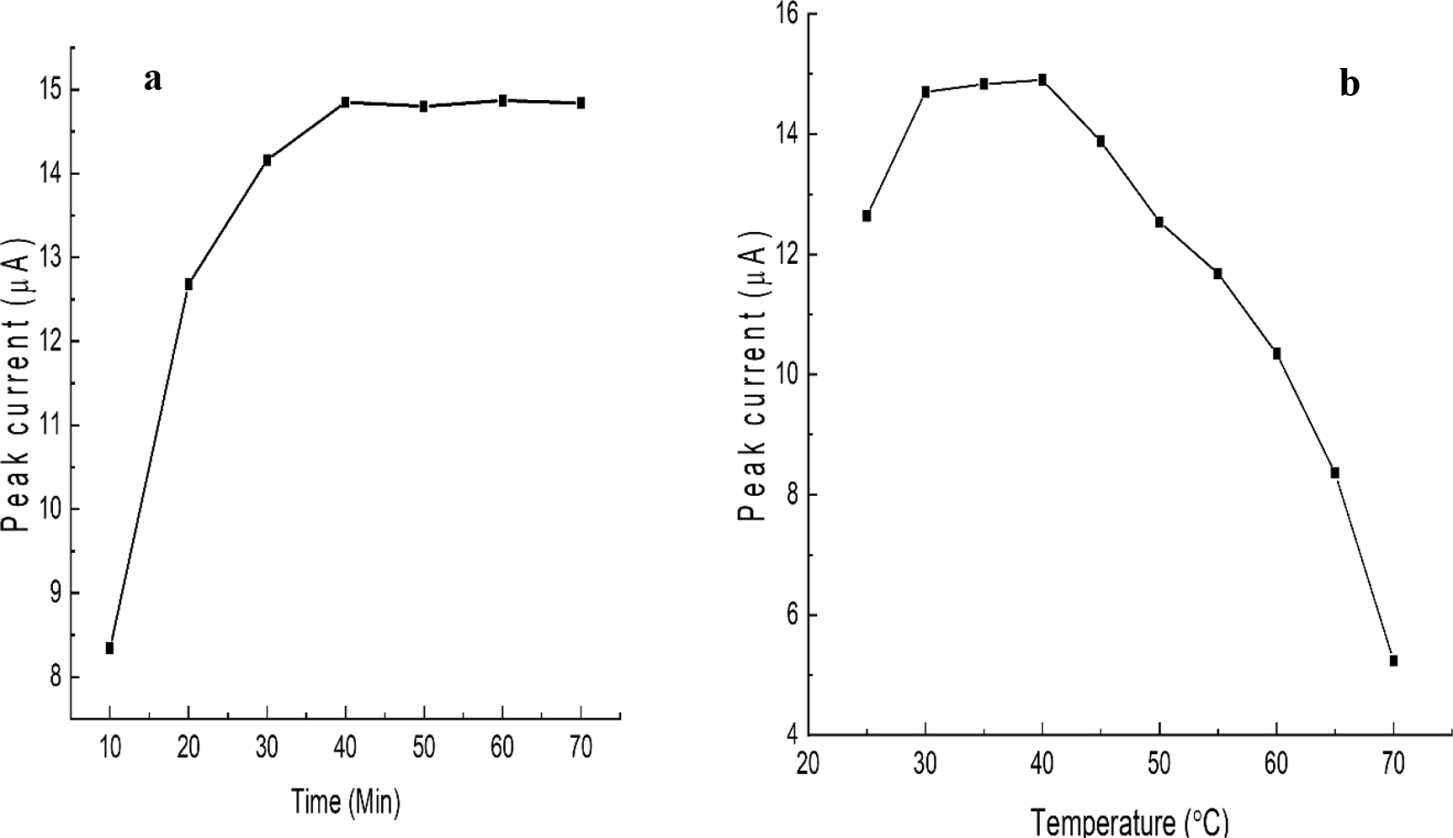
Electrochemical study for the optimisation of reverse complementary DNA hybridisation parameters. (a) Optimisation of hybridisation time (min), and (b) optimisation of hybridisation temperature (°C).

An optimum hybridisation time is required to allow the growth of the duplex when the target DNA is exposed to the immobilised probe. As shown in Figure 4a, 40 min was the best time for hybridisation. After that, the hybridisation state remained stable, and this time was chosen for the subsequent investigations. The hybridisation efficiency linearly increases from 10 to 30 min, following a small increase between 30 and 40 min, and remains almost constant after 40 min until the end (70 min). This indicates that no more hybridisation of reverse complementary DNAs occurs after 40 min. This could be due to the completion of the hybridisation of the reverse complementary DNA with the probe on the electrode surface.

The effect of hybridisation temperature is shown in Figure 4b. The peak current linearly increases up to 30 °C, following an almost slow constant increment between 30 and 40 °C, then it linearly decreases. Therefore, the suitable hybridisation temperature ranges from 30 to 40 °C, although at 40 °C the performance is slightly better. Throughout the trials, the optimal hybridisation temperature was set to 40 °C. Before conducting hybridisation, DNA samples were denatured at 95 °C to make them available as single strands to interact with the corresponding target probe. Unwinding the target DNA and making it available to interact with the immobilised probe DNA requires the right hybridisation temperature, which improves the effectiveness of DNA hybridisation. The hybridisation temperature is affected by a variety of factors, including the length of the DNA sequences, nucleotide base composition (GC content), and concentration of salts used in buffer. Different researchers reported different hybridisation temperatures. Among them, Benvidi et al. [60] reported 35 °C as the hybridisation temperature, whereas Kusnin et al. [57] and Hartati et al. [61] reported 40 °C and room temperature as the hybridisation temperature, respectively. These results are similar to and corroborate our findings.

The pH value is also an important parameter for hybridisation efficiency. In the drop-casting method, there is little scope to conduct experiments at variable pH values due to the use of the microlitre scale solution. Usually, DNA hybridisation efficiency peaks between pH 7.5 and 8.5 due to good accessibility and binding affinity [62]. For this experiment, pH 7.4 was chosen, which is close to physiological pH, and a similar pH value was reported by Kusnin et al. [57] in hybridisation biosensing experiments.

### Selectivity of the electrochemical biosensor

**Figure 5:**
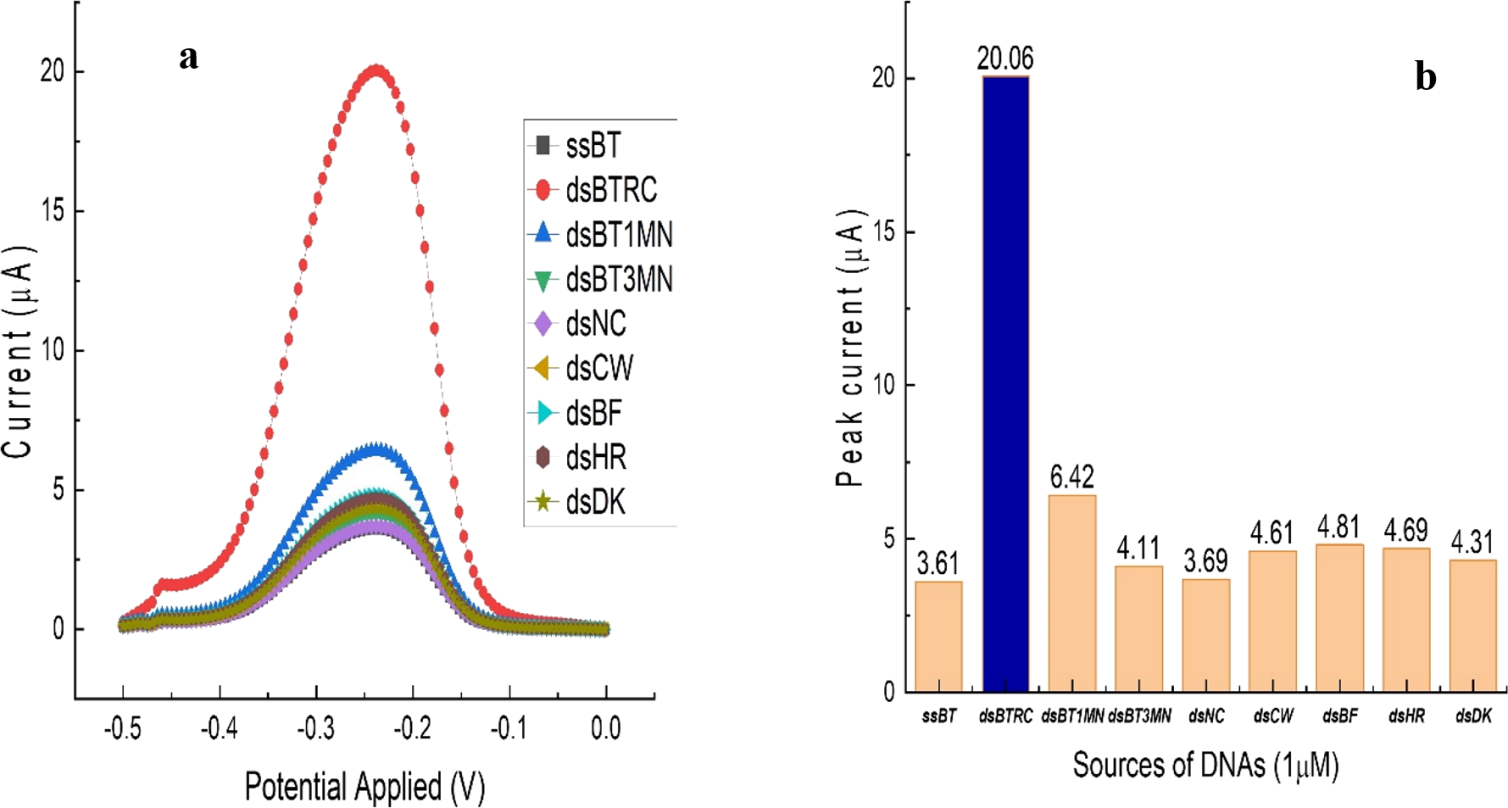
DPV of hybridised DNA in the presence of MB for the RC, different types of mismatch and nontarget species of DNAs in a 10× PB (pH 7.4) solution. (a) DPV voltammogram curves of different DNAs. (b) Peak current histogram of the same DNAs (*n* = 3). ss: single stranded; BT: BT probe; ds: double stranded (to indicate hybridisation state); RC: Reverse complementary; MN: mismatch nucleotide(s) and numbers 1 or 3 correspond to the number of mismatch nucleotides. CW: cow, BF: buffalo, HS: horse, and DK: duck.

Electrodes with hybridised DNA were incubated in MB for 30 min, washed with deionised water, and the efficiency of hybridisation was measured by DPV using MB as a redox species. Figure 5 shows the DPV response to the hybridisation of target, noncomplementary, nontarget, or mismatch DNAs. The DPV current signal for the target DNA is significant and those of the nontarget DNA and the immobilised DNA probe are close to each other, suggesting the selectivity of the target. From a three-base mismatched DNA to a one-base mismatched DNA, peak currents steadily rose. This trend continued with the reverse complementary target. The highest peak current (20.06 μA) was recorded for the reverse complementary target, followed by one-base mismatch reverse complementary (6.42 μA), 3-base mismatch reverse complementary (4.11 μA), and NC (3.69 μA) DNAs. The other nontarget reverse complementary DNA of cow (4.61 μA), buffalo (4.81 μA), horse (4.69 μA), duck (4.31 μA), and probe DNA (3.61 μA) have minimally responded. The peak current and current density of the hybridised nucleotides are shown in Supporting Information File 1, Table S2. The observed pattern of the peak current was that the lowest for noncomplementary DNA, it then gradually increased with the reduction of mismatch in nontarget DNA, and reached the highest peak for the reverse complementary target. This result revealed that the developed DNA biosensor could distinguish between complementary and mismatch DNA with precision.

Research on the application of electrochemical DNA biosensors to detect adulteration has already started. Estrela's group in 2008 reported optimisation of DNA immobilisation on gold electrodes for label-free detection by EIS [63]. Another group published a report for label-free DNA detection using the EIS technique with PNA probes, and they discovered that detection can be greatly improved when using uncharged peptide nucleic acid (PNA) probes [64]. Methylene blue is a very prominent intercalator for DNA-based sensors and biosensors [31,65–69]. For example, Plaxco's group reported the preparation of electrode-immobilised methylene-blue-modified oligonucleotides for electrochemical DNA and aptamer-based biosensing [65]. The Gooding Group published a method for measuring DNA hybridisation by voltammetry on gold electrodes using methylene blue as an intercalator and a self-assembled alkanethiol monolayer. *N*-hydroxysulfosuccinimide (NHS) and *N*-(3-dimethylamino) propyl-*N*-ethylcarbodiimide hydrochloride (EDC) were used to covalently link DNA and oligonucleotides to a carboxylate-terminated alkanethiol self-assembled monolayer (SAM) performed on a gold electrode (AuE) [70]. The Lisdat group described a label-free impedimetric sensor for the detection of hybridisation events which is based on short ssDNA recognition elements [31]. As per our observation, this is the first application of an electrochemical DNA biosensor for BT detection. Kusnin et al. [57] employed silicon nanowires/platinum nanoparticles (SiNWs/PtNPs) on a SPCE for the detection of porcine DNA. The few studies conducted earlier used only one/two random noncomplementary DNA without showing a correlation with the target [57,61,71]. In this study, we used two mismatches, one NC, and four reverse complementary DNAs of four correlated species, which were thoughtfully designed by comparing 29 different species as noncomplementary. The comparison with these has significantly validated the selectivity of the biosensor.

#### Sensitivity of the electrochemical biosensor

**Figure 6:**
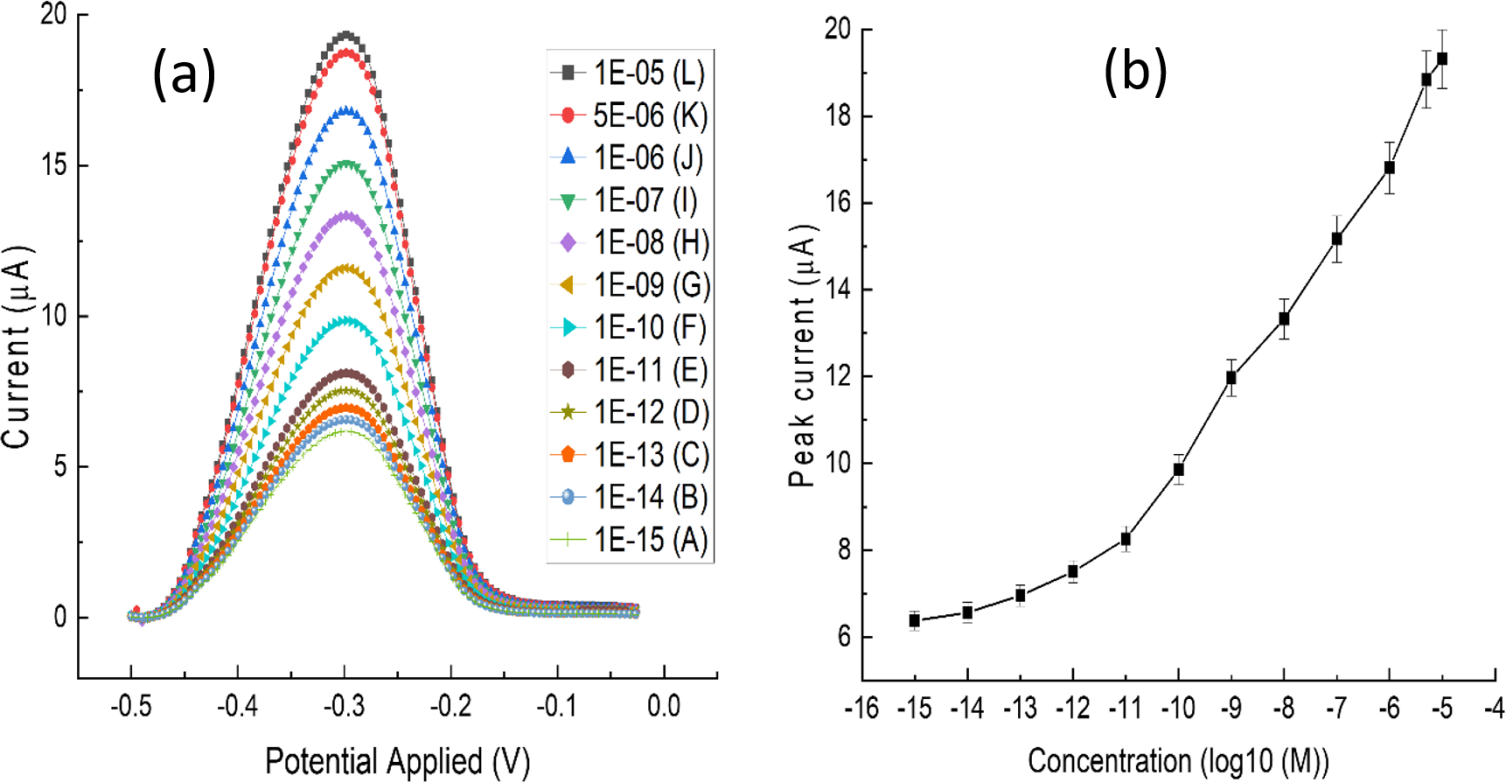
Comparison of the DPV responses of different concentrations of reverse complementary DNA hybridised with BT probe DNA. (a) DPV curve at various concentrations (from 10^−5^ to 10^−15^ M) of reverse complementary DNAs against corresponding values of reverse complementary DNA concentrations, indicated by corresponding different legends and colours. (b) Scattered plot with error bars for the peak current (µA) as a function of the corresponding log concentrations [log_10_(M)] of DNA. All DNA values are at the molar (M) concentration. ‘E’ stands for exponential (power of 10) and (−) stands for negative power.

The sensitivity of the developed electrochemical DNA biosensor was tested against various concentrations of reverse complementary DNA target in the range of 1 × 10^−15^ to 1 × 10^−5^ M. The DPV peak current increased as the reverse complementary DNA concentration increased, as seen in Figure 6a. A calibration curve was constructed using log_10_ concentration of DNA to test the linearity of the DNA concentration with peak current (Figure 6b). The curve exhibited good linearity in the range of 1 × 10^−11^ to 5 × 10^−6^ M. The limit of detection (LOD) of the developed electrochemical DNA biosensor was calculated and found to be 0.85 × 10^−12^ M, which implies that the performance of the developed biosensor is quite satisfactory to detect box turtles in a sample. The previously reported limit of detection ranges from microgram [70] to nanogram [57] levels, which is significantly higher than those of our current findings. Current densities of relative concentrations are presented in Supporting Information File 1, Table S3.

### Reproducibility of the electrochemical biosensor

Reproducibility is the capability of the biosensor to yield identical responses in a recurrent experimental setup. It is a characteristic feature of the precision and accuracy of the biosensor [72] and it is a crucial parameter for the reliability of the application. For the detection of 1 μM of *C. amboinensis* cytb DNA, a series of four probes modified with AuNPs/Gr/SPCE were hybridised with target DNAs. The peak currents within the range of 19.33–20.51 μA were observed when assessed in phosphate buffer (PB), as presented in Table 1. Based on the measured current data, the relative standard deviation (RSD) was calculated using the following equation:


[1] $$RSD=\frac{\sigma \times 100}{\mu },$$

where σ stands for standard deviation and μ stands for the mean of the values. A reasonable RSD of 2.34% was found, which suggests that the developed biosensor has a good reproducibility at a low concentration.

**Table 1:** Reproducibility of the biosensor for 1 µM of target DNA.

Replica	Peak current (μA)	Mean (μ)	Standard deviation (σ)	RSD (%)

1	19.84			
2	20.06	19.94	0.468	2.34
3	20.51			
4	19.33			

### Real sample analysis

**Figure 7:**
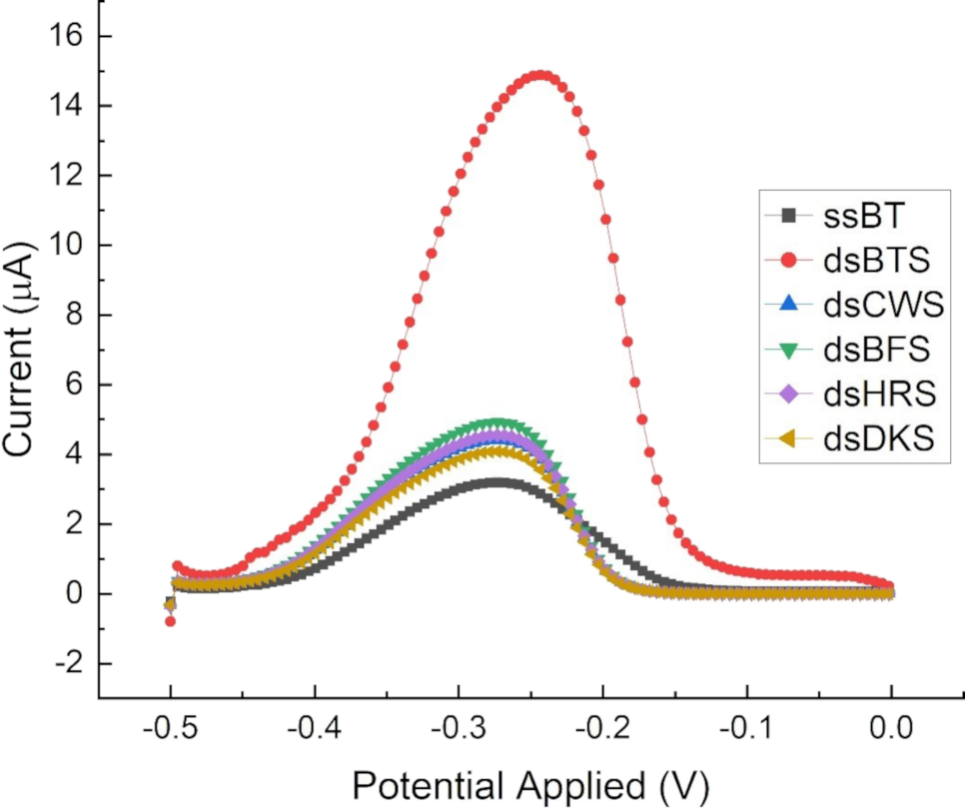
DPV curve of hybridised DNA isolated from raw meat muscle samples. BTS stands for box turtle DNA isolated from a meat sample; ds: double stranded; CWS: cow sample, BFS: buffalo sample, HRS: horse sample, and DKS: duck sample.

The isolated DNA from different muscle samples were hybridised with probe-modified SPCEs. After hybridisation, the selectivity was assayed. Figure 7 shows the DPV response to the hybridised DNAs. The DPV current signal for nontarget DNA species and the immobilised DNA probe are reasonably close to each other, suggesting that only a very small quantity of DNA hybridisation has occurred, whereas turtle DNA showed a significantly higher peak current. This result revealed that the developed DNA biosensor was able to distinguish between turtle DNA and other counterparts with precision. The peak currents and current densities are shown in Supporting Information File 1, Table S4. From the peak currents of the nontarget species, we found that the current response is not negligible compared with the targeted species. Therefore, it may interfere with the reproducibility during real sample analysis.

## Conclusion

The design of an in silico-based *C. amboinensis* cytb gene-specific probe sequence, as well as the use of a nanocomposite on a fabrication platform resulted in a simple, selective, and sensitive electrochemical DNA biosensor for the detection of targeted BT. The utilisation of a AuNPs/Gr nanocomposite enhanced the active surface area and conductivity of the modified SPCE and also enhanced its electrochemical capacity. The attachment of the thiol group to the probe facilitated covalent attachment with AuNPs, and the carbon spacer arm facilitated protrusion from the electrode surface, which enables proper hybridisation. As an intercalator for dsDNA, MB was used. The MB intercalates within Watson–Crick base pairs of dsDNA through a thread-like embolism technique. Thus, the sensing signal is based on the accumulation of MB, which is proportional to the hybridised DNA concentration. Thus, it can easily differentiate between complementary and noncomplementary DNAs to distinguish BT from other nontarget species and intentionally introduce mismatches in the reverse complementary DNAs. On the basis of this approach, we were able to discriminate BT from cow, buffalo, horse, duck, and one/three base mismatch as well as NC DNA sequences using synthetic and real DNA samples by direct experimental evidence. Based on the evidence of sequence alignment and probe design followed by mismatch count, it can be inferred that the biosensor will be able to discriminate BT from other species. The electrochemical DNA biosensor exhibited high sensitivity, excellent selectivity, and good stability with the desired detection limit. It might have a promising future for detecting box turtle adulteration in food products and solving the problem of point-of-care application after commercial adaptation. However, attaining good reproducibility in real samples is always challenging compared to those of laboratory results in PB. Hence, it is still one of the potential barriers to the commercialization of electrochemical biosensors. From that standpoint, there is still room for advancement to reduce the drawbacks associated with reproducibility and precise detection in real samples.

## Experimental

**Figure 8:**
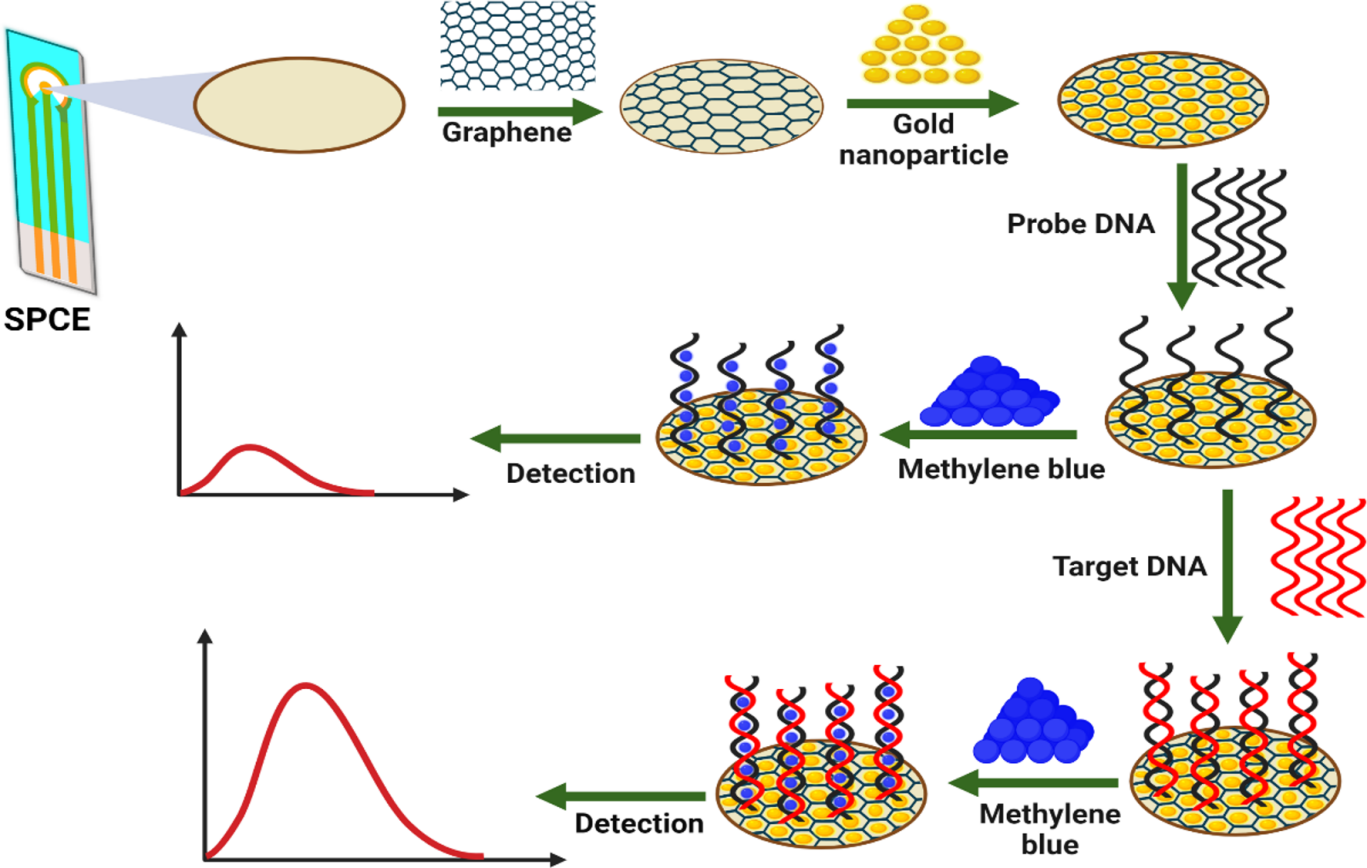
Step-by-step fabrication procedure of the electrochemical DNA biosensor for the detection of the BT cytb gene. Figure 8 was created with BioRender.com. This content is not subject to CC BY 4.0.

The overall scheme of an electrochemical DNA biosensor for the detection of *C. amboinensis* is shown in Figure 8.

### Design of species-specific DNA probe

Mitochondrial cytb gene sequences from BT and other 29 relevant species were retrieved from the NCBI database (https://www.ncbi.nlm.nih.gov/) and aligned using the MEGA5.2 [73] sequence alignment software followed by ClustalW [74] to find intraspecies conserved and interspecies hypervariable regions. The specific DNA sequence of BT was selected as a probe by comparing the BT cytb gene sequence with the same gene of other nontarget species within the aligned sequences having the maximum mismatch with nontarget sequences (Figure 1). Then, the designed probe sequence was submitted to BLAST (basic local alignment search tool) on NCBI to check the in silico specificity by comparing with all sequences in the online database. The designed probe was modified with a thiol modifier to facilitate covalent attachment with AuNPs on the modified electrode surface, and a six-carbon spacer arm was added to facilitate extrusion from the electrode surface. The designed DNA sequences were synthesised from GENEWIZ (China) with the following sequence (the underlined bases indicate the number of mismatches with the probe sequence).

Thiolated ssDNA probe (ssBT): HS-S-(CH_2_)_6_-5′-GATCATTACTAGGCACCTGCCTAATCCTTCA-3′

Reverse complementary (RC) ssDNA: 5′-TGAAGGATTAGGCAGGTGCCTAGTAATGATC-3′

RC one base mismatch ssDNA (1MN): 5′-TGAAGGATTAGGCAAGTGCCTAGTAATGATC-3′

RC three base mismatch ssDNA (3MN): 5′-TGAAGGATTAGGTGAGTGCCTAGTAATGATC-3′

Cow RC ssDNA (CW): 5′-TGTAGGATTAGGCAGATTCCCAGGAGGGAAC-3′

Buffalo RC ssDNA (BF): 5′-TGCAGGATTAGGCAGATGCCTAGGAGAGAGC-3′

Horse RC ssDNA (GT): 5′-TGGAGGATTAGGCAGATTCCTAGGAGGGAGC-3′

Duck RC ssDNA (CK): 5′-TGTAGAATCAAGCATACTCCTAGTAAGGATC-3′

Non complementary (NC): 5′**-**CAGGAAGCCGAATGAACATTCGACGGCAGCT**-**3′

### Management and storage of the probe and other synthetic DNA sequences

The lyophilised DNA powder (obtained from the manufacturer) was processed and diluted with autoclaved deionised water to make a 100 μM stock. The stock and diluted solutions were kept at −40 °C. For the preparation of a 10 μM reduced probe solution, 100 μL of the 100 μM thiol-modified probe solution was placed into 850 μL of autoclaved deionised water and mixed well. Then, 50 μL of 20 mM TCEP was added to the solution and thoroughly mixed, followed by incubation at room temperature for 2 h to complete the reduction process.

### Pretreatment and modification of screen-printed carbon electrodes with the Gr/AuNPs nanocomposite

Before modification, the bare SPCE surface was cleaned with ten CV cycles in a 0.1 M H_2_SO_4_ solution at a scan rate of 50 mV/s. After that, the electrode was entirely washed with ultrapure water, then ultrasonically cleaned in ultrapure water, and dried in an oven at 50 °C for 2 h. Graphere was dispersed into deionised water by ultrasonication to form a Gr dispersion with a 1 mg/mL concentration. A volume of 8 μL of the Gr suspension was carefully dropped onto the SPCE surface to obtain Gr/SPCE. Similarly, 8 μL of AuNPs was drop-cast three times to another piece of SPCE and denoted as AuNPs/SPCE. Both were air-dried at room temperature for 1 h. Again, AuNPs were dropped three times onto the Gr/SPCE surface, which was then labelled as AuNPs/Gr/SPCE.

### Morphological and structural studies of nanomaterials

The surface morphology of the nanoparticles and their nanocomposites were analysed using a field-emission scanning electron microscope (model SU8030 Hitachi, Japan), equipped with an Oxford Instrument energy dispersive X-ray spectrometer. UV–vis was performed on a Libra S80 Biochrom spectrophotometer and FTIR was performed on a Spectrum 400 PerkinElmer spectrometer (U.S.).

### Electrochemical studies of the electrodes

The electrochemical behaviour of different modified SPCE surfaces was analysed with a Metrohm autolab potentiostat/galvanostat utilising the NOVA 2.1.4 software. The modified SPCE was electrochemically characterised using CV, DPV, and EIS at a scan rate of 100 mV/s vs Ag/AgCl as the reference electrode in the potential range of −0.1 to 0.6 V. A solution of 2 mM potassium ferricyanide was used as a redox indicator in a 0.2 M KCl buffer at room temperature.

### Immobilisation of the capture probe

The AuNPs/Gr/SPCE was used as an immobilisation platform for the predesigned single-stranded DNA probe (ssBT). A volume of 8 μL of the ssBT solution at a concentration of 1 µM was dropped onto the surface of AuNPs/Gr/SPCE and allowed to dry at room temperature. Next, the surface of AuNPs/Gr/SPCE was rinsed with a 0.5% SDS solution three times to remove unbound DNAs and then three times with ultrapure water. The electrode was denoted as ssBT/AuNPs/Gr/SPCE.

### Hybridisation of target DNAs

A volume of 8 μL of a solution containing 1.0 μM of the desired DNAs was mixed well with 2.0 μL of 10× SSC buffer by micropipetting on a piece of Parafilm. The mixture was dropped onto the surface of the ssBT/AuNPs/Gr/SPCE. It was allowed to hybridise for 40 min at room temperature and then it was rinsed with 0.5% SDS and ultrapure water remove non-hybridised DNAs. The modified electrode was denoted as dsDNA/AuNPs/Gr/SPCE.

### Electrochemical detection of DNAs using methylene blue

Methylene blue is a redox compound which binds to hybridised dsDNA. Methylene blue acts as a redox species based on which the hybridisation rate/amount is estimated. We performed DPV in the absence and after incubation with MB. The modified electrodes, ssBT/AuNPs/Gr/SPCE, and dsDNA/AuNPs/Gr/SPCE were immersed into 10× PB containing 2 × 10^−5^ mol/L of MB, incubated for 30 min, and then rinsed with deionised water three times. The electrochemical detection was carried out in a potential range of −0.5– 0 V at a scan rate of 100 mV/s in a 10× PB solution using DPV. The current intensity was not observed in the absence of MB.

### Optimisation of experimental conditions

Two major parameters (i.e., hybridisation time and temperature) were studied based on changes in the DPV response of the hybridised DNA intercalating with MB at pH 7.4, and were optimised to enhance hybridisation efficiency. The effect of each parameter was studied by varying the values within a specific range while holding the other parameters constant. For this purpose, hybridisation times were checked from 10 to 70 min, and temperatures were within 25 and 70 °C.

### Electrochemical measurements

An Autolab potentiostat/galvanostat was used to perform the electrochemical experiments. The modified SPCE was electrochemically characterised using CV, DPV, and EIS at a scan rate of 100 mV/s vs a Ag/AgCl electrode in the potential range of −0.1–0.6 V. The DPV was carried out to identify the hybridisation of DNAs at a scan rate of 100 mV/s in the potential range from −0.5–0 V. All measurements and analysis were run utilising the NOVA 2.1.4 software. The effective surface area was determined using CV at a scan rate ranging from 40 to 150 mV/s. The EIS analysis was performed at 0.20 V as the applied potential and at a frequency of 0.1–100 kHz with a 10 mV amplitude. The DPV was carried out with a step potential of 5.0 mV and modulation amplitude of 25 mV. All the tests were performed at room temperature.

#### Selectivity, sensitivity, and reproducibility of the biosensor

The hybridisation process between the immobilised DNA probe and the target, mismatch, noncomplementary, or nontarget DNAs was carried out as described in the ‘Hybridisation of target DNAs’ subsection. Hybridised electrodes were incubated in MB for 30 min, washed with deionised water, and the efficiency of hybridisation was measured by DPV using MB as the redox species. Various concentrations of reverse complementary DNA in the range of 1 × 10^−15^ (A) to 1 × 10^−5^ M (L) were used in sensitivity analysis to determine the efficiency and limit of detection of the fabricated electrode. The reproducibility of the biosensor was obtained by hybridisation of four separate ssBT/AuNPs/Gr/SPCEs using 8 μL of 1 μM of BT cytb reverse complementary DNA sequence.

### DNA extraction from raw meats and detection

Total DNA from meats was isolated using a DNA extraction kit (Favorgen Biotech Corp, Taiwan) following manufacturer instructions. A UV–vis spectrophotometer (NanoPhotometer Pearl, Implen GmbH, Germany) was used to assess the extracted DNA content and purity at an absorbance of 260 nm and at an absorbance ratio of A260/A280 [19]. The extracted DNA was heated at 95 °C for 8 min in the PCR machine (ABI Veriti, Applied Biosystems) to denature the dsDNA into ssDNA. It was then immediately dropped onto an SPCE-modified probe and detected by using DPV as stated in previous sections.

## Supporting Information

All data that support the findings of this study are included within the article.

File 1 Materials, methods and tables.
