# Supplementary material for: Rapid and sensitive detection of box turtles using an electrochemical DNA biosensor based on a gold/graphene nanocomposite
Source: Beilstein J Nanotechnol. 2022 Dec 6;13:1458–72. doi: 10.3762/bjnano.13.120 (PMC9749552; doi:10.3762/bjnano.13.120)
Supplement: File 1 — Materials, methods and tables. [file Beilstein_J_Nanotechnol-13-1458-s001.pdf]

BEILSTEIN JOURNAL OF NANOTECHNOLOGY

Supporting Information

for

Rapid and sensitive detection of box turtles using an electrochemical DNA biosensor based on a gold/graphene nanocomposite

Abu Hashem, M. A. Motalib Hossain, Ab Rahman Marlinda, Mohammad Al Mamun, Khanom Simarani and Mohd Rafie Johan

Beilstein J. Nanotechnol. **2022,** *13,* 1458–1472. doi:10.3762/bjnano.13.120

Materials, methods and tables

License and Terms: This is a supporting information file under the terms of the Creative Commons Attribution License (https://creativecommons.org/licenses/by/4.0). Please note that the reuse, redistribution and reproduction in particular requires that the author(s) and source are credited and that individual graphics may be subject to special legal provisions.
The license is subject to the *Beilstein Journal of Nanotechnology* terms and conditions: (https://www.beilstein-journals.org/bjnano/terms)

Materials and Methods

Materials

All the chemicals were of analytical grade. NaOH, KCl, KNO_3, $K_4[Fe(CN)_6]$, H_2SO_4, HCl, $Na_2HPO_4.7H_2O$, KH_2PO_4, $NaH_2PO_4.H_2O$ were purchased from Merck (Germany). NaCl was purchased from John Kollin (USA) and Na_2HPO_4 and trisodium citrate from Systerm (Malaysia). Graphene was synthesised in this laboratory and AuNPs were purchased from Cytodiagnostics (Canada). Tris (2-carboxyethyl) phosphine (TCEP) was purchased from Acros Organics, Thermo Fisher scientific (Malaysia). All aqueous solutions were prepared with 18.2 MΩ cm ultra-pure water from PURELAB Classic (ELGA VEOLIA, UK).

Apparatus

Cyclic voltammetry (CV), DPV, and electrochemical impedance spectroscopy (EIS) were conducted by using the Metrohm μAutolab potentiostat/galvanostat with the NOVA 2.1.4 software (Metrohm, Switzerland). SPCE was collected from Metrohm (Switzerland). Other equipment used were a pH metre from Sartorius (Germany), a microcentrifuge from Sigma (Germany), an analytical balance from Merck (Germany), and a stirrer from PMC (Barnstead/Thermolyne, USA).

Table S1: Comparison of FTIR peaks for different nanoparticles and their composites.

Materials	Major peaks (at cm^{-1})										
Gr	3734	2357 (downward)	2165	1964	1561	1397	1162	957	737	679	613
AuNPs/Gr	3704	2299 (downward)	2159	1986	1586	1411	1149	920	721	671	606
DNA/AuNPs/Gr	3721	2310 (upward)	2019	1922 (almost disappear)	1569	1413 (Almost disappear)	1254 (disappear)	disappear	795	721	626

Table S2: Peak current and current density of the hybridised DNAs based on DPV.

Sl.	Nucleotide sources	Peak current (μA)	Peak current density (μA/cm^{2})
1	ssBT	3.61	32.82
2	dsBTRC	20.06	182.36
3	dsPK1MN	6.42	58.36
4	dsPK3MN	4.11	37.36
5	dsNC	3.69	33.55
6	dsCW	4.61	41.91
7	dsBF	4.81	43.73
8	dsHR	4.69	42.64
9	dsDK	4.31	39.18

Table S3: Peak current and current density for sensitivity analysis based on DPV results.

Sl.	Nucleotide (concentration)	Peak current (μA)	Peak current density (μA/cm^{2})
A	1×10^{-15}	6.38	58.00
B	1×10^{-14}	6.57	59.73
C	1×10^{-13}	6.96	63.27
D	1×10^{-12}	7.51	68.27
E	1×10^{-11}	8.27	75.18
F	1×10^{-10}	9.86	89.64
G	1×10^{-9}	11.98	108.91
H	1×10^{-8}	13.33	121.18
I	1×10^{-7}	15.17	137.91
J	1×10^{-6}	16.81	152.82
K	5×10^{-6}	18.84	171.27
L	1×10^{-5}	19.32	175.64

Table S4: Peak current and current density for real samples related to BT.

Sl.	Samples	Peak current (μA)	Peak current density (μA/cm$^{2)}$
1	ssBT	3.2	29.09
2	dsBTS	14.88	135.27
3	dsCWS	4.41	40.09
4	dsBFS	4.93	44.82
5	dsHRS	4.55	41.36
6	dsDKS	4.09	37.18
